# Direct oral anticoagulants (DOAC) for prevention of recurrent arterial or venous thromboembolic events (ATE/VTE) in myeloproliferative neoplasms

**DOI:** 10.1007/s00277-020-04350-6

**Published:** 2020-11-20

**Authors:** Karlo Huenerbein, Parvis Sadjadian, Tatjana Becker, Vera Kolatzki, Eva Deventer, Carina Engelhardt, Martin Griesshammer, Kai Wille

**Affiliations:** 1 University Clinic for Hematology, Oncology, Hemostaseology and Palliative Care, Johannes Wesling Medical Center Minden, UKRUB, University of Bochum, Hans-Nolte-Straße 1, D-32429 Minden, Germany; 2 Statistical Consulting and Data analysis, Hameln, Germany

**Keywords:** Myeloproliferative neoplasms, Direct oral anticoagulants, Thrombosis, Anticoagulation therapy, Bleeding

## Abstract

In patients with *BCR-ABL*-negative myeloproliferative neoplasms (MPN), arterial or venous thromboembolic events (ATE/VTE) are a major burden. In order to control these complications, vitamin K antagonists (VKA) are widely used. There is no robust evidence supporting the use of direct oral anticoagulants (DOAC) in MPN patients. We therefore compared the efficacy and safety of both anticoagulants in 71 cases from a cohort of 782 MPN patients. Seventy-one of 782 MPN patients (9.1%) had ATE/VTE with nine ATE (12.7%) and 62 VTE (87.3%). Forty-five of 71 ATE/VTE (63.4%) were treated with VKA and 26 (36.6%) with DOAC. The duration of anticoagulation therapy (*p* = 0.984), the number of patients receiving additional aspirin (*p* = 1.0), and the proportion of patients receiving cytoreductive therapy (*p* = 0.807) did not differ significantly between the VKA and DOAC groups. During anticoagulation therapy, significantly more relapses occurred under VKA (*n* = 16) compared to DOAC treatment (*n* = 0, *p* = 0.0003). However, during the entire observation period of median 3.2 years (0.1–20.4), ATE/VTE relapse-free survival (*p* = 0.2) did not differ significantly between the two anticoagulants. For all bleeding events (*p* = 0.516) or major bleeding (*p* = 1.0), no significant differences were observed between VKA and DOAC. In our experience, the use of DOAC was as effective and safe as VKA, possibly even potentially beneficial with a lower number of recurrences and no increased risk for bleedings. However, further and larger studies are required before DOAC can be routinely used in MPN patients.

## Introduction

In patients with *BCR-ABL*-negative myeloproliferative neoplasms (MPN), arterial and venous thromboembolic events (ATE/VTE) occur frequently and have a significant impact on morbidity and mortality [1–3]. Several studies reported up to 10 times higher incidence of such complications compared to the healthy population [4–12].

Vitamin K antagonists (VKA) are widely used for both primary therapy and secondary prevention of MPN-associated ATE/VTE [13–15]. The introduction of direct oral anticoagulants (DOAC) provides a new treatment option [14, 16–20] with data comparable to the VKA [16–21]. However, the DOAC have no drug approval for patients with cancer or hematological malignancies, and published experience with its use in MPN-associated vascular events is currently very limited.

Curto-Garcia et al. [22] retrospectively reported on the results of 32 MPN patients with 38 venous thromboembolism and DOAC treatment. During a median follow-up period of 2.1 years, neither VTE recurrences nor major bleedings were observed.

Ianotto et al. [21] reported on the retrospective course of 25 MPN patients under DOAC treatment. During a median follow-up time of 2.1 years, two arterial thromboembolic and three bleeding events were observed. A case-control comparison of 25 MPN patients treated with low-dose aspirin (ASS) showed no difference in efficacy and safety [21].

Preliminary and retrospective data from Fedorov et al. [23] reported 22 DOAC- and 31 VKA-treated MPN patients with comparable incidences of recurrence and bleeding events.

However, there is no robust evidence supporting the use of DOAC in MPN, including a direct comparison with VKA treatment. Therefore, at our center, we retrospectively evaluated 71 MPN patients with 71 ATE/VTE treated with VKA (*n* = 45) or DOAC (*n* = 26) to compare the efficacy and safety of both anticoagulants.

## Patients and methods

Clinical data of all MPN patients, who regularly present in our university department, were collected from June 2007 to April 2020 (time of last data cut-off April 1, 2020). All MPN were diagnosed according to the WHO 2008 criteria [24–26]. A total of 782 MPN patients (478 female, 61.1%, and 304 males, 38.9%) are currently registered in our outpatient clinic specializing in MPN. The median age is 50.5 years (range: 11.0–88.9), and the median follow-up time is 1.8 years (range: 0.1–28.4). The different MPN subtypes within the whole group are essential thrombocythemia (ET), *n* = 254 (32.5%); polycythemia vera (PV), *n* = 264 (33.8%); myelofibrosis (MF) including primary and secondary myelofibrosis, *n* = 238 (30.4%); and MPN unclassifiable, *n* = 26 (3.3%). The driver mutations are distributed within the 782 MPN patients as follows: *JAK2* mutation, 534 (68.3%); *CALR* mutation, 110 (14.1%); *MPL* mutation, 19 (2.4%); triple negative, 42 (5.4%); and unknown 70 (9.0%).

The main objective of this retrospective, non-interventional, single-center study was to compare VKA with DOAC therapy in MPN patients. Of particular interest was the efficacy in ATE/VTE treatment, the prevention of ATE/VTE relapses, and the subsequent risk of bleeding complications under both anticoagulants. The data were collected in an electronic system. The Ethics Committee of our center approved the study. We focused on each patient with at least one MPN-associated arterial (ATE) or venous (VTE) thromboembolic event treated with VKA or DOAC. In line with previous studies, we defined an ATE or VTE associated with MPN if it occurred within 2 years prior to MPN diagnosis or after [27, 28].

The follow-up time was defined as the time between the first occurrence of an ATE/VTE and last visit to our center. Treatment time was defined as the time between the start of anticoagulation (= after the first ATE/VTE) and the end of anticoagulation or the last visit to our center (if anticoagulation was not stopped) or first relapse (whichever came first).

For each MPN patient with an ATE/VTE and anticoagulation with VKA or DOAC, we collected demographic data, mutational profile, method of objective diagnosis of ATE/VTE, and presence of cardiovascular (CV) risk factors. In addition, further details on ATE/VTE such as localization, total number, PT (prothrombin time)-INR (international normalized ratio), time of diagnosis and other cytoreductive or antiplatelet therapy were collected. Cytoreductive treatment was defined as the use of hydroxyurea, busulfan, anagrelide, interferon-alpha, ruxolitinib, and/or other JAK inhibitors at the time of ATE/VTE or within 6 months thereafter. Finally, time intervals regarding anticoagulation treatment, duration of treatment, occurrence of bleeding complications, and the number of relapses that occurred during or after completion of anticoagulation were recorded. The diagnosis of an ATE/VTE event required objective diagnostic procedures such as ultrasound, computed tomography, angiography, or scintigraphy.

The severity of bleeding complications was defined according to the criteria of the International Society on Thrombosis and Hemostasis [29]. According to these criteria, we considered a bleeding greater than II° (e.g., transfusion-related anemia, central nervous system involvement, or other life-threatening bleeding) to be clinically relevant.

## Statistical methods

For continuous variables, the median and range are provided. The annual incidence of ATE/VTE recurrences was calculated by dividing the number of events by the sum of patient-years. Differences in the proportions were estimated using Fisher’s exact test, Chi-square test, Mann-Whitney *U* test (statistical significance threshold set at *p* < 0.05), or log-rank test (Mantel-Haenszel test).

## Results

Of the total 71 MPN-associated ATE/VTE, nine (12.7%) were ATE and 62 (87.3%) VTE. Table 1 provides an overview of demographic data and clinical characteristics of all 71 patients diagnosed with 71 initial ATE/VTE. Most ATE/VTE patients were female (*n* = 49, 69.0%) and were diagnosed as PV (*n* = 30, 42.3%), followed by MF (*n* = 20, 28.2%) or ET (*n* = 19, 26.8%). Two MPN patients with ATE/VTE were found to have a MPN at bone marrow biopsy, but both could not be further classified and were referred to as MPN unclassifiable. The *JAK2* V617F mutation was the most frequent driver mutation (*n* = 63, 88.7%). The median age at ATE/VTE diagnosis was 54.0 years (22.0–82.0).

**Table 1:** Overview of demographic data and clinical features of 71 MPN patients with 71 arterial and venous thromboembolic events (ATE/VTE) treated with either VKA or DOAC

Male/female; *n* (%)	22/49 (31.0/69.0)
Median age at first ATE/VTE; years (range)	54.0 (22.0–82.0)
Median follow-up time from first ATE/VTE; years (range)	3.2 (0.1–20.4)
MPN diagnosis	
Polycythemia vera (PV); *n* (%)	30 (42.3)
Myelofibrosis (MF); *n* (%)	20 (28.2)
Essential thrombocythemia (ET); *n* (%)	19 (26.8)
MPN unclassifiable; *n* (%)	2 (2.8)
Driver mutations	
*JAK2*; *n* (%)	63 (88.7)
*CALR*; *n* (%)	3 (4.2)
*MPL*; *n* (%)	1 (1.4)
Triple negative; *n* (%)	2 (2.8)
Unknown; *n* (%)	2 (2.8)
ATE/VTE	
VTE; *n* (%)	62 (87.3)
ATE; *n* (%)	9 (12.7)

The localizations of all 71 ATE/VTE events together with corresponding localizations of ATE/VTE in VKA- or DOAC-treated MPN patients are shown in Table 2. Most ATE (6/9 = 67%) consisted of transient ischemic attacks (*n* = 2) or a stroke (*n* = 4). The remaining three had two embolisms each on a lower limb (*n* = 2, 2.8%) and an arterial splenic infarction (*n* = 1, 1.4%). About half VTE (28/62 = 45%) were atypical with 22 splanchnic and six sinus vein thromboses. Deep vein thrombosis simultaneously with pulmonary embolism was observed in nine MPN patients (*n* = 9, 12.7%). Isolated deep vein thrombosis or pulmonary embolism occurred in 14 (19.7%) and nine (*n* = 9, 12.7%) MPN cases with VTE, respectively. The two remaining VTE were each thrombophlebitis (*n* = 1, 1.4%) and arm vein thromboses (*n* = 1, 1.4%).

**Table 2:** Localization of all first arterial and venous thromboembolic events (ATE/VTE, *n* = 71) with corresponding localizations of ATE/VTE in DOAC (*n* = 26) or VKA (*n* = 45) treated MPN patients together with localization of first ATE/VTE recurrences (*n* = 26)

	First ATE/VTE event (*n* = 71)	First ATE/VTE treated with DOAC (*n* = 26)	First ATE/VTE treated with VKA (*n =* 45)	ATE/VTE recurrences (*n* = 26)	ATE/VTE recurrences after DOAC therapy (*n* = 4)	ATE/VTE recurrence during or after VKA therapy (*n* = 22)
Localization						
Arterial thromboembolic events (ATE); *n* (%)	9 (12.7)	3 (11.5)	6 (13.3)	12 (46.2)	1 (25.0)	11 (50.0)
Transient ischemic attack (TIA); *n* (%)	2 (2.8)	-	2 (4.4)	1 (3.8)	-	1 (4.5)
Angina pectoris; *n* (%)	-	-	-	1 (3.8)	-	1 (4.5)
Stroke; *n* (%)	4 (5.6)	1 (3.8)	3 (6.7)	2 (7.7)	-	2 (9.1)
Arterial embolism of lower limb; *n* (%)	2 (2.8)	1 (3.8)	1 (2.2)	3 (11.5)	-	3 (13.6)
Renal infarction; *n* (%)	-	-	-	1 (3.8)	1 (25.0)	-
Splenic infarction; *n* (%)	1 (1.4)	1 (3.8)	-	4 (15.4)	-	4 (18.2)
Venous thromboembolic events (VTE)	62 (87.3)	23 (88.5%)	39 (86.7)	14 (53.8)	3 (75.0)	11 (50.0)
Deep vein thrombosis; *n* (%)	14 (19.7)	5 (19.2)	9 (20.0)	6 (23.1)	1 (25.0)	5 (22.7)
Pulmonary embolism; *n* (%)	9 (12.7)	6 (23.1)	3 (6.7)	1 (3.8)	1 (25.0)	-
Deep vein thrombosis simultaneous to pulmonary embolism; *n* (%)	9 (12.7)	4 (15.4)	5 (11.1)	-	-	-
Splanchnic vein thrombosis; *n* (%)	22 (31.0)	5 (19.2)	17 (37.8)	4 (15.4)	-	4 (18.2)
Thrombophlebitis; *n* (%)	1 (1.4)	1 (3.8)	-	1 (3.8)	1 (25.0)	-
Sinus vein thrombosis; *n* (%)	6 (8.5)	2 (7.7)	4 (8.9)	2 (7.7)	-	2 (9.1)
Arm vein thrombosis; *n* (%)	1 (1.4)	-	-	-	-	-

No recurrences were observed during DOAC therapy, but there were four recurrences after stopping DOAC. 22 recurrences occurred during or after VKA therapy

All 71 patients had a median total follow-up time of 3.2 years (range: 0.1–20.4) with an incidence rate for all 71 ATE/VTE of 3.4% per patient/year. The corresponding rates for ATE and for VTE were 0.4% and 3.0% per patient/year, respectively.

Out of 71 MPN with a first ATE/VTE, 45 (63.4%) were treated with VKA and 26 (36.6%) with DOAC. Most patients with DOAC received rivaroxaban (*n* = 21), and the remaining were treated with apixaban (*n* = 5). The median duration on anticoagulation was 1.0 years (range 0.1–20.4) with a median time on VKA of 1.0 years (range 0.1–20.4) and a median time on DOAC of 1.3 years (range 0.2–7.3). During the entire anticoagulation period, low-dose acetylsalicylic acid was additionally used in the 71 ATE/VTE in seven patients with VKA therapy (7/45, 9.9%) and in four patients with DOAC treatment (4/26, 5.5%). Cytoreductive treatment was initiated in 39 MPN patients (39/71, 63.9%) simultaneously or within 6 months after ATE/VTE. In the VKA group 22 of 45 patients (48.9%) and in the DOAC group 17 of 26 patients (65.4%) additionally received cytoreductive drugs.

**Fig. 1:**
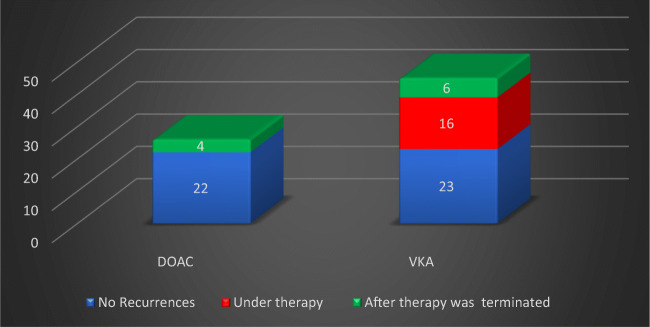
First ATE/VTE recurrences (*n* = 26) during follow-up time: significantly more recurrences (*p* = 0.0003) occurred during VKA (*n* = 16) compared to no recurrences during DOAC (*n* = 0) treatment (red). After termination of anticoagulation, four of 26 DOAC and six of 45 VKA-treated patients had ATE/VTE recurrences (green). Overall, significantly more recurrences were recorded in patients with VKA treatment (*n* = 22) compared to DOAC (*n* = 4) (*p* = 0.0053)

Within a median time of 1.5 years (range: 0.1–8.5), 26 first ATE/VTE recurrences were observed in 26 patients. This corresponds to an ATE /VTE recurrence rate of 8.0% per patient/year. No recurrence was observed in 45 patients (63.4%). The localizations of the first 26 recurrences together with the corresponding localizations of recurrent ATE/VTE in VKA- or DOAC-treated MPN patients are shown in Table 2. Of 26 first recurrences, 12 (12/26 = 46.2%) were ATE and 14 (14/26 = 53.8%) were VTE. After a median time of 0.9 years (range: 0.1–8.5), 16 ATE/VTE recurrences occurred during anticoagulation with VKA therapy (16/26 = 61.5%). No recurrences were observed during DOAC therapy. This difference is statistically significant (*p* = 0.0003) (Fig. 1).

In 17 out of 45 patients (38%) treated with VKA, sufficient PT (prothrombin time) and/or INR (international normalized ratio) values were documented during anticoagulation, and 53% of these were in the therapeutic range. At the time of relapse, seven out of 22 patients treated with VKA had documented PT and/or INR values, and four (57%) were in the therapeutic range.

During the entire follow-up time, 22 recurrences occurred in VKA-treated (*n* = 22, 84.6%) and four in DOAC-treated (*n* = 4, 15.4%) patients. In the latter group, all four recurrences were recorded within a median time of 0.7 years (range: 0.3–1.3) after termination of DOAC treatment. Sixteen of all 22 VKA-associated ATE/VTE recurrences (16/22 = 72.7%) were observed during VKA anticoagulation therapy. The remaining six recurrences (6/22 = 27.3%) were observed within a median time of 0.9 years (range: 0.4–4.0) after termination of the VKA. Comparing the total number of recurrences in VKA-treated patients (*n* = 22) with the recurrences registered in patients treated with DOAC (*n* = 4) shows a statistically significant difference during the follow-up time (*p* = 0.0053) (Fig. 1).

**Fig. 2:**
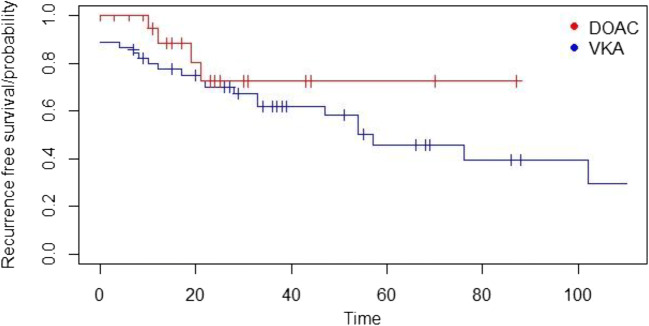
Probability of recurrence-free survival: the cumulative probability of the ATE/VTE recurrence-free survival in 71 MPN patients treated with DOAC (*n* = 26, red curve) or VKA (*n* = 45, blue curve) was statistically not significantly different (*p = 0.2*)

After comparing the absolute number of ATE/VTE recurrences, an analysis was performed that considered the probability of “recurrence-free” survival during the follow-up time. In this analysis, the difference between VKA- and DOAC-treated patients was not statistically different (Fig. 2, *p = 0.2*). The incidence rate of ATE/VTE recurrences in VKA-treated patients was 8.1% per patient/year and 7.2% per patient/year in DOAC-treated patients. This difference was also not statistically different (alpha = 5%).

## Bleedings

During anticoagulation with either VKA or DOAC, 10 of 71 patients (14.1%) experienced 11 bleeding complications over a median period of 1.6 years (range: 0.1–6.8). Six out of 11 bleedings (54.5%) were classified as severe bleedings.

In the VKA group, seven bleeding complications (63.6%), including four major bleeding complications, were recorded after a median time of 1.6 years (range: 0.1–6.8). Three out of four major bleedings (one esophageal varicose vein bleeding and two severe epistaxis episodes) occurred during VKA use alone (without low-dose acetylsalicylic acid). One patient underwent a combination therapy of VKA and low-dose acetylsalicylic acid and experienced major postoperative bleeding 1 day after total hip replacement implantation. During VKA treatment, three minor bleedings occurred (a menorrhagia, an episode with bloody semen, and an unspecified bleeding tendency).

During DOAC therapy, two minor and two major bleeding complications (*n* = 4, 36.4%) occurred after a median time of 0.5 years (range: 0.3–1.6). Both major bleeding episodes under DOAC anticoagulation (without low-dose acetylsalicylic acid) were gastrointestinal bleedings of unknown localization. The remaining two minor bleedings were epistaxis and petechial bleeding during DOAC therapy.

Overall, no significant differences were observed between DOAC and VKA anticoagulation therapy for both overall (*p* = 0.516) or major bleeding (*p* = 1.0). A comparison regarding different clinical and laboratory parameters between VKA- and DOAC-treated patients is shown in Table 3. In the VKA group, only the total follow-up time (*p* = 0.0005) and number of ATE/VTE recurrences (*p* = 0.0053) were statistically different.

**Table 3:** Comparison of different clinical and laboratory parameters between DOAC- (*n* = 26) and VKA-treated MPN patients (*n* = 45)

Parameters	DOAC	VKA	*p*
Number of pts.*	26	45	
Median age at MPN diagnosis; years (range)	55.5 (24.0–81.0)	50.0 (22.0–82.0)	0.131
Median age at first ATE/VTE event; years (range)	57.5 (27.0–88.0)	53.0 (22.0–81.0)	0.070
Gender (male/female)	8/18	14/31	0.976
Essential thrombocythemia	8	11	0.816
Polycythemia vera	10	20	
Myelofibrosis	7	13	
*JAK2 Mutation*	24	39	0.701
Cardiovascular risk factors (yes/no)	15/11	30/15	0.450
ATE	3	6	1.0
VTE	23	39	
Median treatment time; years (range)	1.3 (0.2–7.3)	1.0 (0.1–20.4)	0.984
Median total follow up time; years (range)	1.7 (0.2–7.3)	4.8 (0.6–20.4)	0.0005**
ATE/VTE recurrences	4	22	0.0053**
Combined ASS use***	4	7	1.0
Cytoreductive therapy for first ATE/VTE****	17	22	0.22
Bleeding events total	4	7	1.0
Major bleeding events	2	4	1.0
Deaths	1	3	1.0

*Pts. = patients

**Significantly different

***During time on anticoagulation after first ATE/VTE

****Begin at time of ATE/VTE or within 6 months thereafter

## Discussion

Myeloproliferative neoplasm (MPN) patients have an increased risk of arterial and venous thromboembolic events (ATE/VTE). In larger MPN cohorts, the proportion of patients suffering from ATE/VTE is reported to be 10 to 30% [22]. Accordingly, vascular events occurred in 9.1% (71/782) of our 782 MPN patients. The incidence rate for the first 71 ATE/VTE was 3.4% per patient/year with a VTE rate of 3.0% per patient/year. Prospective studies in MPN observed comparable VTE rates of 0.5–3.7% [6, 7]. The ATE incidence rate of 0.4% per patient/year in our MPN patients was also similar to the reported ATE rates of 0.2 to 1.5% [5, 11].

In recent decades, anticoagulation with vitamin K antagonists (VKA) has been the treatment of choice to prevent ATE/VTE recurrences in MPN patients. Hernández-Boluda et al. [14] reported a 2.8-fold risk reduction for recurrence in 150 ET and PV patients with ATE/VTE and VKA treatment. In 206 MPN patients, De Stefano et al. [15] also found a reduction in the recurrence rate of VTE with VKA. The incidence rate of recurrent VTE was 5.3% per patient/year among patients with long-term VKA and 12.8% per patient/year after VKA discontinuation (*p* = 0.008). The VTE recurrence rate in our cohort was comparable at 8.0% per patient/year.

As far as anticoagulation with direct oral anticoagulants (DOAC) is concerned, there are few studies in MPN that indicate good efficacy with sufficient safety. In a retrospective study by Curto-Garcia et al. [22] in 32 MPN patients receiving DOAC for MPN-associated venous thromboembolism treated with DOAC, no VTE relapse but one ATE occurred. No major and three minor bleedings were reported. Ianotto et al. [21] retrospectively reported two ATE and no VTE recurrences in a cohort of 25 DOAC-treated MPN patients. Three major and two minor bleedings were observed. Curto-Garcia et al. [22] reported a median age of 49.9 years and a median follow-up of 2.1 years in their publication. The median follow-up time in the study of Ianotto et al. [21] was quite similar with 2.1 years. However, both studies did not compare DOAC treatment with VKA in their cohort [21, 22]. Fedorov et al. [23] reported preliminary data on recurrence rates and bleeding complications in 22 DOAC- and 31 VKA-treated MPN patients. During a short follow-up of 8 months, the number of ATE/VTE recurrences (DOAC, *n* = 5 versus VKA, *n* = 6) and of all bleeding complications (DOAC, *n* = 5 versus VKA, *n* = 11) were not significantly different.

The median age of our 71 MPN patients at the time of ATE/VTE was 54 years and was comparable to the studies of Curto-Garcia et al. and Ianotto et al. [21, 22]. The median duration of anticoagulation was lower at 1.0 years for VKA and 1.3 for DOAC. During anticoagulation therapy, significantly more relapses occurred under VKA (*n* = 16) compared to DOAC treatment (*n* = 0, *p* = 0.0003). However, during the entire observation period of median 3.2 years (0.1–20.4), ATE/VTE relapse-free survival (*p* = 0.2) did not differ significantly between the two anticoagulants. This is mainly due to the significantly longer follow-up time for VKA patients (*p* = 0.0005). The corresponding recurrence rates for VKA and DOAC treatment (during and after discontinuation of anticoagulation) did not differ significantly either.

During anticoagulation with VKA, 53% and at the time of relapse, 57% of patients treated with VKA were in the therapeutic range with PT-INR. A major disadvantage of the VKA is the narrow therapeutic range and the time patients spend in the therapeutic range (TTR, “time in therapeutic range”). Even in well-conducted comparative studies of VKA and DOAK, the TTR was on average only between 55 and 65% [17, 20, 30, 31].

As in the studies mentioned above, we have not observed any increased bleeding propensity under DOAC. In particular, the rate of major bleeding was not higher under DOAC compared to VKA. Regarding bleeding complications with anticoagulation, the German MPN Registry of the Leukemia Study Alliance [32] reported bleeding in 437 MPN patients, including eight with DOAC (rivaroxaban) treatment. In a multivariate analysis, the risk of bleeding during DOAC treatment was slightly reduced compared to VKA.

In summary, our results complement the currently limited literature [21–23, 32] on the efficacy and safety of DOAC-treated MPN patients. Despite the limitations—small number of patients, retrospective analysis, and short treatment time—our data suggest that the use of DOAC was as effective and safe as VKA. However, further and larger studies are required before DOAC can be routinely used in MPN patients.

## Data Availability

The datasets generated during and/or analyzed during the current study are available from the corresponding author on reasonable request.
